# Neurofeedback training improves anxiety trait and depressive symptom in GAD

**DOI:** 10.1002/brb3.2024

**Published:** 2021-01-27

**Authors:** Yue Hou, Shuqin Zhang, Ning Li, Zhaoyang Huang, Li Wang, Yuping Wang

**Affiliations:** ^1^ Department of Neurology Xuanwu Hospital Capital Medical University Beijing China; ^2^ The Beijing Key Laboratory of Neuromodulation Beijing China; ^3^ Center of Epilepsy Beijing Institute for Brain Disorders Capital Medical University Beijing China

**Keywords:** eeg biofeedback, generalized anxiety disorder, increase of alpha brain wave amplitude, neurofeedback

## Abstract

**Objective:**

To investigate the effectiveness of alpha activity neurofeedback training over the parietal lobe in GAD patients.

**Methods:**

Twenty‐six female patients who had been diagnosed as GAD according to the Diagnostic and Statistical Manual of Mental Disorders (5th edition, DSM‐V) criteria were included in this study. Patients were randomized into two groups: the left parietal lobe training group (LPL group, *n* = 13) and the right parietal lobe training group (RPL group, *n* = 13), and then received ten 40‐minute alpha training sessions in the relevant area. Evaluations included severity of anxiety (by State‐Trait Anxiety Inventory, STAI) and depression (by Beck Depression Inventory, BDI‐II) after the fifth training session and the last training session.

**Results:**

The scores of STAI‐S decreased significantly two weeks after the fifth training session in both groups (LPL group: from 47.15 ± 10.65 to 38.69 ± 8.78, *p*<.05; RPL group: from 44.92 ± 12.37 to 37.31 ± 6.41, *p* < .05) and decreased further at the four weeks’ time point after the last training session (LPL group: 35.15 ± 9.24; RPL group: 29.85 ± 6.18). Compared with baseline, the scores of STAI‐T, BDI‐II and ISI decrease at two weeks, no significant difference found between LPL group and RPL group. The scores of STAI‐T, BDI‐II and ISI decreased at four weeks when compared with two weeks, and no significant difference found between LPL group and RPL group.

**Conclusion:**

Neurofeedback training of alpha activity over the parietal lobe is effective in GAD patients, especially the anxiety trait and depressive symptoms.

## Graphical abstract

Neurofeedback training of alpha activity over the parietal lobe is effective in GAD patients. Especially on the improvement of GAD patients’ anxiety trait and depressive symptoms.

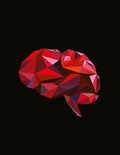

## 1 INTRODUCTION

Generalized anxiety disorder (GAD) is one of the most common psychological disorders. The combined lifetime prevalence of GAD was 3.7%, 12‐month prevalence was 1.8%, and 30‐day prevalence was 0.8% (Ruscio et al., 2017) . Lifetime prevalence estimates varied widely across countries, ranging from less than 1% to approximately 8% of the populations (Ruscio et al., 2017). GAD is characterized by intense anxiety and worry regarding several events or activities that persist most days during at least six months and is difficult to control (American Psychiatric Association, 2013). Treatment options for GAD generally include pharmacological therapies or psychological therapies. However, not all patients respond to these therapies and some patients may experience adverse effects (Stein & Sareen, 2015). Cognitive behavioral therapy (CBT) is the most well studied and commonly used in all of the psychological methods. But CBT cannot be used widespread enough in China because of the relative shortage of doctors and long‐term follow‐up. (Li et al., 2017) Therefore, additional research of another kind of psychological and more easily to operate strategies to improve GAD treatment is needed.

Biofeedback (BF) is a noninvasive psychophysiological treatment technique with a bio‐monitoring system and sensors to measure, amplify, and feedback information that enables an individual to learn how to change physiological activity and thus improve health and performance (Schoenberg & David, 2014). Neurofeedback as a specific type of biofeedback focuses on the brain to improve neuroregulation and stabilization (Fovet et al., 2015; Marzbani et al., 2016). Modulation of brain activity can affect behavioral changes (Micoulaud‐Franchi et al., 2015).The results from one RCT (Dadashi et al., 2015) suggested that NF may be effective for the treatment of GAD compared with no treatment.

Alpha is the dominant EEG rhythm in healthy adults at rest and is associated with a calm, relaxed state (Stinson & Arthur, 2013; Watson et al., 1979). Tim Lomas et al. conducted a systematic review of EEG studies of mindfulness meditation. Mindfulness was associated with enhanced alpha power and elevated alpha may signify a state of relaxed alertness (Lomas et al., 2015). It has long been understood that anxiety disorders are associated with physiological arousal (Bond et al., 1974; Hoehn‐Saric & McLeod, 1988). Anxiety‐related arousal can be detected centrally using electroencephalography (EEG), with some evidence that attenuated alpha activity is associated with anxiety (Wise et al., 2011). Increasing alpha magnitude can produce a calming effect in high‐anxious individuals (Hardt & Kamiya, 1978). Frontal alpha asymmetry is assumed to be associated with psychopathology and individual differences in emotional responding (Tolin et al., 2020). In the recent past (Dias & Deusen, 2011; Kerson et al., 2009; Wang et al., 2013), induction of healthy alpha asymmetry and regulation of alpha power bands have been successfully used to treat anxiety and depression. Neurofeedback is a tool that can be used to change frontal alpha asymmetry and could prove to be a practical intervention option to increase resilience.

Previous studies found that patients with GAD have attentional bias to threatening and negative stimuli (Amir et al., 2009; Armstrong et al., 2011; Mogg et al., 2000; Waters et al., 2008). And the attention networks have been implicated to contribute to attentional bias, which is thought to contribute to the pathophysiological mechanisms of GAD (Bar‐Haim et al., 2007). Megan et al. found behavioral improvement was unrelated to reliance on the perceptual network but positively related to reliance on the attentional network, that is frontoparietal attention network (deBettencourt et al., 2015). Regions along the dorsal areas of the parietal cortex, including the superior parietal lobule (SPL) and the intraparietal sulcus (IPS), are involved in top‐down attentional orienting, while ventral regions including the temporo‐parietal junction (TPJ) are involved in bottom‐up attentional orienting (Benjamin Hutchinson et al., 2009). Parietal cortex plays important roles in attention networks (Benjamin Hutchinson et al., 2009; Vossel et al., 2014). Additionally, D Scheinost et al. found increased control over anxiety was associated with decreased connectivity in the orbitofrontal cortex and increased connectivity in a right parietal region (Scheinost et al., 2013). Brambilla et al. found that white‐matter connectivity is impaired in the right parietal lobe in patients with GAD (Brambilla et al., 2012). July et al. use neurofeedback protocol to improve a female patient’s mild anxiety and sleep quality, and found alpha changes from the pretreatment baseline were particularly prominent at P4 (Gomes et al., 2016). These findings suggested that right parietal lobe might play an important role in the pathophysiological mechanisms of GAD.

In light of these considerations, the present study aimed to confirm the effectiveness of alpha‐increase neurofeedback training over the parietal lobe in GAD and compare the effects of the left parietal lobe (LPL) training and the right parietal lobe (RPL) training.

## 2 METHODS

This study was a randomized controlled open‐label study with two groups. Twenty‐six female patients were enrolled and randomly assigned to LPL training group (*n* = 13) and RPL training group (*n* = 13). All the patients of the study were informed and the written consent of the patients was obtained. This study was approved by the Ethics Committee of Xuanwu Hospital.

### 2.1 Patients

Twenty‐six female with GAD were recruited from June 2017 to December 2018 in Beijing, China, Xuanwu Hospital, Capital Medical University according to Protocol CRR 2020024. Diagnosis of GAD was according to the Diagnostic and Statistical Manual of Mental Disorders (5th edition, DSM‐V) criteria (American Psychiatric Association, 2013).

Inclusion Criteria: (a) Diagnosis of GAD by DSM‐V; (b) ≥18 years and nonperimenopausal; (c) Right‐handed.

Exclusion Criteria: (a) Sedative hypnotics, antidepressants, or anxiolytics less than 4 weeks; (b) Patients with psychotic disorders, substance‐related disorders, mental retardation; (c) Abnormal laboratory tests of Liver function and Renal function; (d) Pregnancy; (e) Unwillingness to sign the ICF.

The demographic characteristics and baseline evaluation scores of the study subjects are shown in Table 1.

**TABLE 1:** Patient characteristics and baseline scores

	LPL group	RPL group	t	*p*
	Mean ± *SD*	Mean ± *SD*		
*n*	13	13	‐‐‐	‐‐‐
Age (years)	32.6 ± 8.5	32.8 ± 9.0	−0.045	.965
BMI	22.1 ± 2.3	21.5 ± 3.1	0.512	.613
Duration	21.2 ± 13.3	21.3 ± 15.9	−0.013	.989
Treatment	4/9	2/11	*	**
STAI‐S	47.15 ± 10.65	44.92 ± 12.37	0.493	.627
STAI‐T	51.62 ± 9.91	47.77 ± 7.47	1.117	.275
BDI‐II	20.23 ± 10.47	17.69 ± 7.24	0.719	.479
ISI	17.46 ± 5.33	15.46 ± 6.86	0.830	.415

BMI, Body Mass Index; BMI, Kg/m^2^; STAI‐S, State Anxiety Inventory‐S; STAI‐T, State‐Trait Anxiety Inventory‐T; BDI‐II, Beck Depression Inventory –II; ISI, Insomnia Severity Index.

* *x* ^2^ = 0.867;^**^ *p* = .645>.05

### 2.2 Neurofeedback training

Each training session began with a 5‐minutes baseline recording at rest with eyes closed, in order to establish the baseline alpha power score. Mean baseline values were used to calculate the threshold for the training session, defined as mean activity +0.85 standard deviations. Three 7‐minutes neurofeedback training trials followed, with a 2‐minutes inter‐trial break. Patients in the left neurofeedback group received positive feedback if they increased relative P3 alpha power. Patients in the right neurofeedback group received positive feedback if they increased relative P4 alpha power. Patients were provided with a visual and audio feedback consisting of a histogram reflecting the current alpha power. The visual and audio feedback via the computer screen to present in front of the patient that they can seeing and listening “real‐time.” That is, when the alpha power was exceeded the threshold (i.e., desired state), the histogram was green and they could seeing and listening the videos continuously; if the alpha power below the threshold, the histogram instantly turned red and the videos pause. Finally, another 5‐min baseline monitoring of alpha power after each training.

The total NFB training for every patient includes ten times training like above within two weeks. And each patient was asked to do the similar practice 1–2 times per day at home without any biofeedback instrument.

Evaluations included severity of anxiety (by State‐Trait Anxiety Inventory, STAI), depression (by Beck Depression Inventory, BDI‐II) and insomnia (by Insomnia Severity Index, ISI) at baseline, after the fifth training session and after the tenth training session.

### 2.3 Statistical analysis

All statistical analyses were conducted by SPSS 22.0 (SPSS Inc.,).The age, BMI, course of disease, baseline scores (STAI‐S, STAI‐T, BDI‐II, ISI) of enrolled patients were performed by Normal test. The scores of STAI‐S, STAI‐T, BDI‐II, ISI in two groups (LRL and RPL) at baseline, after fifth session and after tenth session were performed by repeated measure ANOVA. The baseline EEG α activity of the two groups (LPL, RPL) was compared by repeated measurement ANOVA. A *p* value less than .05 was considered with statistically significant difference.

## 3 RESULTS

The scores of STAI‐S, STAI‐T, BDI‐II, and ISI at baseline, two weeks and four weeks after treatment in each group described in Table 2.

**TABLE 2:** Comparison of STAI‐S, STAI‐T, BDI‐II, ISI at Baseline, two weeks and four weeks after treatment

		Baseline	Two weeks	Four weeks
STAI‐S	LPL	47.15 ± 10.65	38.69 ± 8.78	35.15 ± 9.24
	RPL	44.92 ± 12.37	37.31 ± 6.41	29.85 ± 6.18
	Before%	‐‐‐	17.94%	25.45%
		‐‐‐	16.94%	33.55%
STAI‐T	LPL	51.62 ± 9.91	46.23 ± 8.05	42.69 ± 9.38
	RPL	47.77 ± 7.47	40.92 ± 6.42	36.92 ± 6.90
	Before%	‐‐‐	10.44%	17.30%
		‐‐‐	14.34%	22.71%
BDI‐II	LPL	20.23 ± 10.47	14.54 ± 8.83	12.08 ± 7.33
	RPL	17.69±7.24	14.08 ± 6.71	10.31 ± 5.98
	Before%	‐‐‐	28.13%	40.29%
ISI		‐‐‐	20.41%	41.72%
	LPL	17.46 ± 5.33	13.00 ± 5.16	8.38 ± 4.72
	RPL	15.46 ± 6.86	10.15 ± 5.08	6.85 ± 3.11
	Before%	‐‐‐	25.54%	52.00%
		‐‐‐	34.35%	55.69%

Before%: ( score at Baseline – score after two weeks treatment or score after four weeks treatment)/ score before treatment × 100%

### 3.1 STAI‐S score

According to Shapiro–Wilk test, the data of each group obeyed normal distribution (*p* > .05); the data were expressed in the form of mean ± standard deviation (Table 2). In LPL training group, STAI‐S scores at baseline, 2 weeks after treatment and 4 weeks after treatment were 47.15 ± 10.65, 38.69 ± 8.78, and 35.15 ± 9.24, respectively. The STAI‐S scores of RPL training group at baseline, 2 weeks after treatment and 4 weeks after treatment were 44.92 ± 12.37, 37.31 ± 6.41, and 29.85 ± 6.18, respectively.

According to Mauchly's spherical hypothesis test, the variance covariance matrix of STAI‐S is equal, *X^2^* = 1.740, *p* = .419.

The results of one‐way ANOVA showed that the interaction term *F* (2,48) = 0.747, *p* = .479 in time ^*^group. The main effect of group *F* (1,24) = 0.959, *p* = .337. Partial Eta squared = 0.038. There was no significant difference in STAI‐S scores among different groups. The main effect of time was *F* (2,48) = 32.506, *p* < .001, partial Eta squared = 0.575. The difference of SAI score at baseline, 2 weeks after treatment and 4 weeks after treatment was statistically significant.

Bonferroni correction method was used for pairwise comparison at three time points (Table 3). The results showed that the difference of STAI‐S scores among the three time points was statistically significant: compared with baseline, the STAI‐S score decreased by 8.04 points at 2 weeks after treatment (*p* = .001, 95% CI: 3.20, 12.87); compared with 2 weeks after treatment, STAI‐S scores of 4 weeks after treatment decreased by 5.50 points (*p* = .003, 95% CI: 1.69, *p* = .003), compared with baseline,STAI‐S score decreased by 13.54 points at 4 weeks after treatment (*p* < .001, 95% CI: 9.21, 17.87).

**TABLE 3:** Comparison of four scores at three time points (paired comparison)

	Baseline vs after Two weeks(*n* = 13)				Baseline vs after Four weeks(*n* = 13)				After Two weeks vs after Four weeks(*n* = 13)			
	Mean Difference	*p*	95% Confidence Interval		Mean Difference	*p*	95% Confidence Interval		Mean Difference	*p*	95% Confidence Interval	
			Upper Limit	Lower Limit			Upper Limit	Lower Limit			Upper Limit	Lower Limit
STAI‐S	8.04	.001	3.20	12.87	13.54	<.001	9.21	17.87	5.50	.003	1.69	9.31
STAI‐T	6.12	.001	2.26	9.97	9.89	<.001	6.86	12.91	3.77	.039	0.15	7.38
BDI‐II	4.65	.001	1.80	7.51	7.77	<.001	4.98	10.56	3.12	.007	.75	5.49
ISI	4.89	<.001	3.09	6.68	8.85	<.001	5.93	11.76	3.96	<.001	1.80	6.13

### 3.2 STAI‐T score

According to Shapiro–Wilk test, the data of each group obeyed normal distribution (*p* > .05); the data were expressed in the form of mean ± standard deviation (Table 2). The STAI‐T scores of LPL training group at baseline, 2 weeks after treatment and 4 weeks after treatment were 51.62 ± 9.91, 46.23 ± 8.05, and 42.69 ± 9.38, respectively; in RPL training group, the STAI‐T scores at baseline, 2 weeks after treatment and 4 weeks after treatment were 47.77 ± 7.47, 40.92 ± 9.38, and 36.92 ± 6.90, respectively.

According to Mauchly's spherical hypothesis test, the variance covariance matrix of STAI‐T is equal, *X^2^* = 1.769, *p* = .413

The results of one‐way ANOVA showed that the interaction term *F* (2,48) = 0.270, *p* = .764 in time * group. The main effect of group was *F* (1,24) = 3.231, *p* = .085. Partial Eta squared = 0.119. There was no significant difference in STAI‐T scores among different groups. The main effect of time was *F* (2,48) = 26.676, *p* < .001, partial Eta squared = 0.526. The difference of STAI‐T score at baseline, 2 weeks after treatment and 4 weeks after treatment were statistically significant.

Bonferroni correction method was used to make a pairwise comparison at three time points (Table 3). The results showed that the difference of STAI‐T scores among the three time points was statistically significant: compared with baseline, the STAI‐T score decreased by 6.12 points at 2 weeks after treatment (*p* = .001, 95% CI: 2.26, 9.97); compared with 2 weeks after treatment, STAI‐T scores of 4 weeks after treatment decreased by 3.77 points (*p* = .039, 95% CI: 0.15, *p* = .001), compared with baseline, the STAI‐T score decreased by 9.89 points at 4 weeks after treatment (*p* < .001, 95% CI: 6.86, 12.91).

### 3.3 BDI‐II score

According to Shapiro–Wilk test, the data of each group obeyed normal distribution (*p* > .05); the data were expressed in the form of mean ± standard deviation (Table 2). In LPL training group, BDI‐II scores at baseline, 2 weeks after treatment and 4 weeks after treatment were 20.23 ± 10.47, 14.54 ± 8.83, and 12.08 ± 7.33; in RPL training group, BDI‐II scores at baseline, 2 weeks after treatment and 4 weeks after treatment were 17.69 ± 7.24, 14.08 ± 7.33, and 10.31 ± 5.98, respectively.

The variance covariance matrix of BDI‐II was equal by Mauchly's spherical hypothesis test, *X^2^* = 1.152, *p* = .562.

The results of one‐way ANOVA showed that the interaction term *F* (2,48) = 0.507, *p* = .605 in time * group. The main effect of group was *F* (1,24) = 0.310, *p* = .583. Partial Eta squared = 0.013. There was no significant difference in BDI‐II scores among different groups. The main effect of time was *F* (2,48) = 28.138, *p* < .001, partial Eta squared = 0.540. The BDI‐II scores before treatment, 2 weeks and 4 weeks after treatment were statistically significant.

Bonferroni correction method was used to compare the BDI‐II scores at three time points (Table 3). The results showed that the BDI‐II scores between the three time points were statistically significant: compared with baseline, the BDI‐II score decreased by 4.65 points (*p* = .001, 95% CI: 1.80, 7.51); compared with 2 weeks after treatment, the BDI‐II scores of 4 weeks after treatment decreased by 3.12 points (*p* = .007, 95% CI: 0.75, *p* = .001), compared with baseline, BDI‐II score decreased by 7.77 points at 4 weeks after treatment (*p* < .001, 95% CI: 4.98, 10.56).

### 3.4 ISI score

According to Shapiro–Wilk test, the data of each group obeyed normal distribution (*p* > .05); the data were expressed in the form of mean ± standard deviation (Table 2). ISI scores of LPL training group at baseline, 2 weeks after treatment and 4 weeks after treatment were 17.46 ± 5.33, 13.00 ± 5.16, and 8.38 ± 4.72, respectively; the ISI scores of RPL training group at baseline, 2 weeks after treatment and 4 weeks after treatment were 15.46 ± 6.86, 10.15 ± 5.08, and 6.85 ± 3.11, respectively.

The variance covariance matrix of ISI is not equal, *X^2^* = 9.285, *p* = .010. Multivariate tests of within subjects effects showed that *F* (2,23) = 32.00, *p* < .001, *F* (2,23) = 0.504, *p* = .610 for time * group. The results showed that the interaction term of time * group (1.501, 36.032) = 0.267, *p* = .703, and the interaction term had no significant effect on the dependent variable. The main effect of group was *F* (1,24) = 1.509, *p* = .231. Partial Eta squared = 0.059. There was no significant difference in ISI score among different groups. The main effect *F* (1.501, 36.032) of time was 47.616, *p* < .001, and partial ETA squared = 0.665. The ISI scores at baseline, 2 weeks, and 4 weeks after treatment were statistically significant.

Bonferroni correction method was used to compare the ISI scores at three time points (Table 3). The results showed that the ISI scores between the three time points were statistically significant: compared with baseline, the ISI score decreased by 4.89 points (*p* < .001, 95% CI: 3.09, 6.68); compared with 2 weeks after treatment, the ISI scores of 4 weeks after treatment decreased by 3.96 points (*p* < .001, 95% CI: 1.80, *p* < .001), compared with baseline, ISI score decreased by 8.85 points at 4 weeks after treatment (*p* < .001, 95% CI: 5.93, 11.76).

## 4 DISCUSSION

For STAI‐S, STAI‐T, BDI‐II, and ISI scores, we tested accordingly. Time factors (*p* < .05) at three time points (before treatment, two weeks of treatment and four weeks of treatment) of the two groups of patients; the grouping factors of the two groups in the left and right treatment groups (*p* > .05); the interaction between time factor and grouping factor (*p* > .05). It means that there are significant differences in the scores of the left treatment group and the right treatment group at three time points, but there is no significant difference in the grouping factors of the two groups, and there is no interaction between the time factors at the three time points and the grouping factors, suggesting the score has a tendency to change with time, but the change in scores does not vary with the group.

Biases in processing threat‐related information played a prominent role in the etiology and maintenance of anxiety disorders (Mathews, 1990; Mathews & Mackintosh, 2000). In other words, the attention system of anxious individuals is distinctively sensitive to a threat‐related stimulus rather than a neutral stimulus in the environment. Several reviews have revealed that this kind of threat‐related attentional bias exists extensively in anxiety disorders such as PTSD (Buckley et al., 2000), social phobia (Clark & McManus, 2002; Heinrichs & Hofmann, 2001; Hirsch & Clark, 2004; Musa & Lépine, 2000), obsessive‐compulsive disorder (OCD) (Summerfeldt & Endler, 1998), GAD (Mogg & Bradley, 2005), and panic disorder and phobias (Mcnally et al., 1999). According to Mansell’s top‐down model (Mansell, 2000) of processing biases in anxiety, attention control is mediated by the anterior cingulate cortex, the lateral prefrontal cortex, and the parietal cortex. Further functional magnetic resonance imaging (fMRI) studies indicated the three attentional networks: (a) alerting network including the classic frontoparietal cortical activation along with the thalamus (Coull et al., 2000; Fan et al., 2005); (b) orienting network including high activity in superior parietal region and the temporal–parietal junction, with a right hemisphere bias (Corbetta et al., 2000; Fan et al., 2005); (c) executive control network including anterior cingulate plus right and left frontal areas activation (Bush et al., 2000; MacDonald et al., 2000). Based on this theory, we conducted neurofeedback training over the parietal lobe and our results confirmed the effectiveness of this method in GAD patients.

Alpha brain wave (8–13 Hz) primarily exists in the occipital lobe during deep relaxation with eyes closed, but not in a tired or asleep condition. Various studies have shown that an increase in EEG alpha wave activity is linked to improved anxiety symptoms (Isotani et al., 2001). After alpha‐increase neurofeedback training, GAD patients’ STAI and BDI‐II scores decreased as expected. Interestingly, attentional bias has also been found in patients with insomnia and attentional bias for sleep‐related negative information is believed to contribute to the mechanism of insomnia (Spiegelhalder et al., 2010).The improvement of insomnia symptoms was positively correlative with the improvement of anxiety symptoms. Our patients’ ISI scores also decreased. Potential mechanism might be that alpha brain wave amplitude training improved GAD patients’ attentional bias through repeated attentional training, thus ameliorating anxiety symptoms.

Compared with high state‐anxiety scores, high trait‐anxiety scores are more difficult to handle. Long‐term follow‐up assessment showed that 6 months after electromyographic biofeedback training, anxiety patients’ state‐anxiety scores remained significantly lower, while trait‐anxiety scores returned to pretreatment levels (Hurley & Meminger, 1992). Our study presented that neurofeedback training of alpha activity over the parietal lobe could improve GAD patients’ anxiety trait. Long‐term follow‐up is ongoing to confirm this result. In addition, there is a high overlap among the anxiety disorders and other mental disorders, for example depression. Most studies identified that the correlation between GAD with major depression was particularly high (Bandelow & Michaelis, 2015; Stein et al., 2017). In our study, high BDI‐II scores were also observed in GAD patients, indicating that they also had some depression symptoms.

Our result was interesting that alpha‐increase neurofeedback training over parietal lobe could also decrease GAD patients’ BDI‐II score.

Moreover, there are limited clinical data regarding the effects of GAD treatment on insomnia symptoms. Although some studies suggested that effective treatment for GAD results in a concomitant improvement in sleep (Uhde et al., 2000), other studies showed that sleep difficulties in GAD often persist after successful treatment of the disorder (Belleville et al., 2010). Many GAD patients’ main complain to hospital is difficult to fall asleep. Our study showed decrease in GAD patients’ ISI score support that insomnia is common in GAD patients.

Though we separate two groups, the fact that only a time effect emerged for the questionnaire and no significant effect emerged from analysis on alpha EEG in any of the groups (LPL, RPL), both groups showed an improvement in anxiety, depression, and insomnia at the end of the training. This just shows neurofeedback training is not like neuromodulation methods (e.g., Repetitive transcranial magnetic stimulation, rTMS). Neurofeedback involves recording information using electrodes placed on the scalp and displaying it, that is feeding it back, on a computer display screen. As the patient alters their own mental state, it changes the amplitudes of various brain wave frequencies. The patient sees this change as it is reflected by various displays on the computer monitor and attempts to alter their brainwave pattern to achieve a predefined goal. In this manner, the patient learns to self‐regulate and the change is the whole brain state. If the EEG area and frequency of brainwave which we observe is suitable, the left or right lateral is not the main effect to influence the results.

There are some limitations in our study. . One main limitation is the fact that only a time effect emerged for the questionnaire, no significant difference in the grouping factors of the two groups. Therefore, many different explanations could underlie the modifications in anxiety or depressive symptoms, such as placebo effects, other treatments, and so on. Second, the sample of this study is small, this might lead to analysis bias in the results. Third, the study subject is limited to female and the follow‐up time is relatively short. Further multicenter research with large sample size and long follow‐up time is needed to prove our conclusion.

## 5 CONCLUSION

Neurofeedback training of alpha activity over the parietal lobe is effective in GAD patients, especially the anxiety trait and depressive symptoms.

## CONFLICT OF INTEREST

There is no conflict of interest in this study.

## **AUTHOR** **CONTRIBUTION**

YH and YW designed the study. YH, SZ, NL and ZH conducted the patient management. LW performed randomization and blinding. YH performed statistical analysis and drafted the article. YW reviewed and revised the article. All authors read and approved the final paper.

### PEER REVIEW

The peer review history for this article is available at https://publons.com/publon/10.1002/brb3.2024.

## Data Availability

All datasets generated for this study are included in the article. All original data can be acquired by contacting correspondence author.
